# The Effectiveness of Brolucizumab and Aflibercept in Patients with Neovascular Age-Related Macular Degeneration

**DOI:** 10.3390/ijerph19042303

**Published:** 2022-02-17

**Authors:** Magdalena Musiał-Kopiejka, Katarzyna Polanowska, Dariusz Dobrowolski, Katarzyna Krysik, Edward Wylęgała, Beniamin Oskar Grabarek, Anita Lyssek-Boroń

**Affiliations:** 1 Trauma Centre, Department of Ophthalmology, St. Barbara Hospital, 41-200 Sosnowiec, Poland; magdamusial73@gmail.com (M.M.-K.); polanowskakatarzyna@gmail.com (K.P.); dardobmd@wp.pl (D.D.); kkrysik@gmail.com (K.K.); 2 Chair and Clinical Department of Ophthalmology, Division of Medical Science in Zabrze, Medical University of Silesia in Katowice, 40-760 Katowice, Poland; ewylegala@sum.edu.pl; 3 Department of Ophthalmology, District Railway Hospital, 40-760 Katowice, Poland; 4 Department of Ophthalmology, Faculty of Medicine in Zabrze, University of Technology, Academy of Silesia in Katowice, 41-800 Zabrze, Poland; 5 Department of Histology, Cytophysiology and Embryology, Faculty of Medicine, University of Technology, Academy of Silesia in Katowice, 41-800 Zabrze, Poland; bgrabarek7@gmail.com

**Keywords:** age-related macular degeneration, retinal pigment epithelium, neovascular age-related macular degeneration, choroidal neovascularization, brolucizumab, aflibercept, VEGF, flow area, select area, FOVEA, visus

## Abstract

Age-related macular degeneration (AMD) is a progressive, chronic disease of the central area of the retina, which, if untreated, leads to blindness. This study aimed to compare the effectiveness of therapy using anti-VEGF drugs, namely brolucizumab and aflibercept, in patients with neovascular AMD (nAMD) during a monitoring period lasting around 20 weeks. The analysis consisted of 40 patients diagnosed with neovascular age-related macular degeneration, with 20 patients receiving aflibercept (Eylea, Bayer) at a dose of 2 mg/50 µL into the vitreous chamber at the following intervals—3 doses, 4 weeks apart, followed by a fourth dose after 8 weeks. The remaining 20 patients received brolucizumab (Beovu, Novartis) at a dose of 6 mg/50 µL, administered in the following schedule—3 initial doses, 4 weeks apart, with the administration of a fourth dose decided for each patient individually by the doctor, depending on disease activity, assessed through imaging tests. To evaluate treatment effectiveness, the following measurements were used: ‘read distance and near visual acuity’ for each eye separately using the Snellen chart; and non-invasive retinal imaging techniques—optical coherence tomography (OCT) and OCT angiography (OCTA). In patients treated using brolucizumab, during the observation period, statistically significant differences were found in the following parameters: flow area (*p* = 0.0277); select area (*p* = 0.0277); FOVEA (*p* = 0.0073); visus (*p* = 0.0064). In brolucizumab-treated patients, changes in OCT and OCTA, indicating an improvement, were already visible after the first injection of the drug, whereas in the aflibercept-treated group, changes were only visible after the fourth injection. We found a higher effectiveness of brolucizumab therapy compared to aflibercept in patients with nAMD during an observations period lasting 20 weeks. Our observations are significant, although they require further research.

## 1. Introduction

Age-related macular degeneration (AMD) is a progressive, chronic disease of the central area of the retina. AMD is the leading cause of blindness in developed nations, currently affecting 8.69% of the world’s population. The incidence of AMD grows with age, and a predicted number of 288 million people will have the disease by 2040 [1].

There are two forms of this disease: the “dry” form, which is the most common (85–90% of patients) and the rarer “wet” form (10–15% of patients); however, it is connected o a higher degree of vision loss [2].

As we better understand the pathogenesis of AMD, there is evidence of overlapping mechanisms existing, highlighting the role of environmental and genetic factors, as well as the many metabolic and functional anomalies of the outer part of the retina, the retinal pigment epithelium (RPE), Bruch’s membrane, and choriocapillaris [2,3,4].

Neovascular AMD (nAMD) is characterized by the presence of abnormal blood vessels known as choroidal neovascularization (CNV), developing as either subretinal or intraretinal [2,5,6].

Under physiological conditions, blood vessel endothelial cells are resistant to neovascular stimuli due to the balance between pro-angiogenic factors, represented by vascular endothelial growth factor (VEGF), and anti-angiogenic factors, such as pigment epithelium-derived factor (PEDF) [7,8].

Pathological conditions, such as hypoxia, ischemia, or inflammation, can tip the balance of angiogenic factors in favor of the formation of new blood vessels [9]. Contemporary researchers suggest that local inflammation plays a role in the initiation and development of choroidal neovascularization (CNV), along with immune responses that create a cellular and molecular environment conducive to pro-angiogenic mechanisms. In fact, neutrophils, macrophages, mast cells and activated microglia can produce and release a variety of factors that promote new vessel formation, in particular VEGF. The final effect of this cascade is the death of photoreceptors due to disturbances in the nutrient supply, retention of morphotic elements extravasated through the walls of abnormal vessels, and scarring [10,11,12,13].

The main aim of treating vascular AMD is the improvement or sustainment of current visual acuity through the inhibition of CNV proliferation and fluid leakage associated with the presence of abnormal vessels [14]. The introduction of anti-VEGF drugs in 2004, which are drugs targeted against VEGF, administered through injection into the vitreous chamber, turned out to be a real a therapeutic revolution. For over 15 years, intravitreal injection of anti-VEGF drugs was the standard of care for the “wet” form of AMD. Currently, four anti-VEGF drugs are available: pegaptanib, bevacizumab, ranibizumab, and aflibercept [15,16]. The effect of anti-VEGF drugs is based on the binding and inactivation of the vascular endothelial growth factor secreted by cells of the retinal pigment epithelium under hypoxic conditions. Drugs directed against VEGF display anti-edemic and anti-inflammatory effects, while also reducing the permeability of the vascular wall and inhibiting angiogenesis [17,18].

Aflibercept is a recombinant fusion protein consisting of fragments of the extra-cellular domains of human VEGF 1 and 2 receptors fused to the FC portion of the human IgG antibody. Aflibercept binds to isoforms A and B of the vascular endothelial growth factor (VEGFA, VEGFB) and placental growth factor (PIGF) forming a stable, neutral complex—which is biologically inactive. After intravitreal administration, aflibercept is slowly absorbed into the circulatory system. In the general circulation, it mainly occurs in the form of an inactive stable complex with VEGF [19,20].

Brolucizumab is a newly developed molecule used to treat nAMD. It is a single-chain variable fragment of the monoclonal Fv antibody, produced through DNA recombination in Escherichia coli cells. Unlike aflibercept, a brolucizumab molecule does not contain the Fc domain of the antibody in its structure, which is responsible for the increased migration of molecules through the blood–retinal barrier. Brolucizumab binds, with high affinity, to VEGFA isoforms, thereby preventing VEGFA from binding to its VEGFR-1 and VEGFR-2 receptors. The small size of the scFV molecule, as well as the lack of the Fc domain, are probably responsible for the greater tissue penetration capacity of brolucizumab, which, in turn results in larger local effectiveness and a more durable end result of brolucizumab treatment [21,22].

This study aimed to compare the effectiveness of anti-VEGF drug therapy, namely brolucizumab and aflibercept, in patients with nAMD during a 20-week monitoring period.

## 2. Materials and Methods

### 2.1. Patients

This study was conducted according to the guidelines of the Declaration of Helsinki. Due to the fact that the drugs used in patients, the effects of which are described in this manuscript, are the treatment standard in Poland (treatment program) and the studies performed in patients did not differ from the accepted standards; this study was retrospective, thus consent of the ethics committee was not required. According to the Polish stature, this is a non-interventional study (Article 37a (1) of the Pharmaceutical Law) and therefore, in the understanding of the Act of 5 December 1996 on the professions of doctor and dentist, does not require the opinion of the bioethical committee and does not constitute clinical trials.

Data confidentiality and patient anonymity were always maintained. Patient identifying information was deleted before the database was analyzed. It is not possible to identify patients on an individual level, either in this article or in the database. Informed conset was obtained from all subjects involved in the study.

Analysis was conducted on 40 patients with diagnosed nAMD. The patients included in this study had no previous treatment in the form of intravitreal injections, so they were treatment naïve. Patients only used topical eye drops to moisturize the surface of the eye, and supplemented with lutein preparations in accordance with the AMD supplementation guidelines. 20 patients (11 women and 9 men, aged 78.33 ± 8.43 years) received aflibercept (Eylea, Bayer) at a dosage of 2 mg, administered in a 0.05 mL solution into the vitreous chamber at the following intervals—3 doses, 4 weeks apart, followed by a fourth dose after 8 weeks. The remaining 20 patients (13 women and 7 men, aged 75.16 ± 3.06 years) received brolucizumab (Beovu, Novartis) at a dosage of 6 mg, administered in a 0.05 mL solution into the vitreous chamber in the following schedule—3 initial doses, 4 weeks apart, with the administration of a fourth dose decided for each patient individually by the doctor, depending on disease activity, assessed through imaging tests. In our study, aflibercept and brolucizumab were used at the doses recommended by the manufacturer, described in the drug charactertistics [23,24,25].

Inclusion criteria to this study, regardless of drug, was as follow:

1. Above 45 years of age;
2. Visual acuity between −0.2 to 0.8 according to the Snellen chart;
3. Presence of an active CNV membrane in the OCT examination using sub-retinal angio-OCT (presence of subretinal and intraretinal fluid);
4. Disk area (DA) change from 3–12;
5. No dominant geographic decline (atrophy);
6. No dominant subretinal hemorrhage;
7. No significant permanent damage to the fovea (atrophy, fibrosis, discoid scar);
8. A patient qualified for aflibercept treatment must be de novo, i.e., not previously treated with intravitreal injections.

Exclusion criteria to this study, regardless of drug, was as follows:

1. Disease progression (decrease in visual acuity which lasts for two consecutive visits);
2. Damage to the central part of the macula;
3. Hypersensitivity to active or auxiliary substances;
4. Dominant subretinal hemorrhage;
5. Active infection of the eye or in its vicinity;
6. Tear retinal detachment or macular hole;
7. Patient qualified for aflibercept treatment previously treated with intravitreal injections.

### 2.2. OCT

To evaluate treatment effectiveness, the following measurements were utilized: long- and short-distance visual acuity measurement for each eye separately, using the Snellen chart from a distance of 5 m for long distance and 35 cm for short distance, the results were expressed according to the Snellen rule; fundus endoscopy using an indirect method with the Volk lens at a focusing power of 90D, after previous pupil dilation using 1% Tropicamid and 10% Neosypherine; non-invasive retinal imaging techniques—optical coherence tomography (OCT) and OCT angiography (OCTA).

OCT examination was conducted using the RTVue (Optovue, Inc, Fremont, California) device, which is a spectral optical tomograph (spectra-domain OCT, SD-OCT), characterized by a quick scanning speed and mapping accuracy of the examined structures. This examination allowed us to visualize scans showing sections of individual layers of the central area at a high resolution, which, furthermore, allowed us to assess the presence of intraretinal and subretinal fluids during active CNV.

### 2.3. OCTA

OCT angiography (Optovue AngioVue^®^, Optovue, Inc.; Freemont, CA, USA) was conducted using a device which utilized the SSADA (split-spectrum amplitude-decorrelation angiography) research algorithm. The retinal macular imaging field in our study was 3 mm × 3 mm. We were able to assess the flow in vessels of the neovascular membrane thanks to technology which uses the reflection of laser light from the surfaces of moving erythrocytes. Using OCT angiography, CNV activity features were assessed, such as: rounded shape changes with a dark “halo” around its perimeter, a branched structure with a dense network of capillaries within it, or the presence of numerous vascular loops. Through the use of the OCTA imaging technique we assessed CNV size and activity before and during anti-VEGF drug treatment.

### 2.4. Statistical Analysis

Statistical analysis was conducted using the licensed version of the STATISTICA 13.3 PL software (Statsoft, Krakow, Poland). All the statistical analyses were conducted using a statistical significance threshold of *p* < 0.05. With the help of the Shapiro–Wilk test, it was determined that non-parametric method should be used for analysis. Therefore, to describe the variables, a median, lower quartile (Q1) and upper quartile (Q3) were used. Furthermore, the Mann–Whitney U test was used to compare the groups of patients qualified for brolucizumab and aflibercept therapy using the flow area, select area, visus and FOVEA variables. To compare two dependent variables, which were flow and select area, before the first and last injection with the given drug, Wilcoxon was used. The ANOVA Friedman test was utilized to conduct statistical analysis of the differences in FOVEA and visual acuity during observation.

## 3. Results

**Figure 1:**
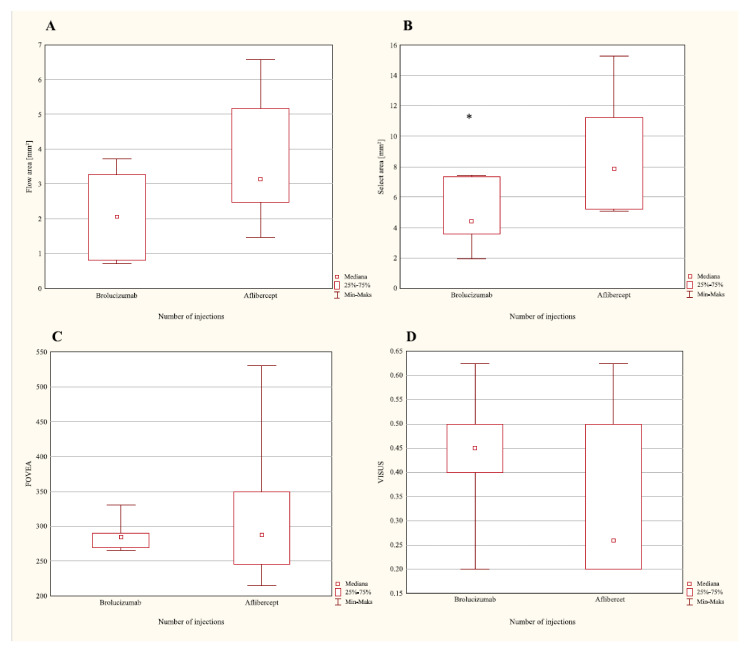
Values of the following parameters: flow area (**A**); select area (**B**); FOVEA (**C**); visus (**D**), among patients with AMD that qualified for brolucizumab and aflibercept therapy, before the first dose of the drug was administered (* *p* < 0.05).

First, the groups of patients qualified for brolucizumab and aflibercept treatment were compared using the parameters: flow area, select area < visus, and FOVEA. The Mann–Whitney U test did not indicate that the differences in flow area, visus, and FOVEA values significantly varied between the groups (respectively, *p* = 0.2403, *p* = 0.3939, and *p* = 0.9327; Figure 1). Only the select area parameter was observed to have a significantly higher value in the aflibercept-treated group, compared to the patients treated using brolucizumab (*p* = 0.0260; Figure 1).

**Figure 2:**
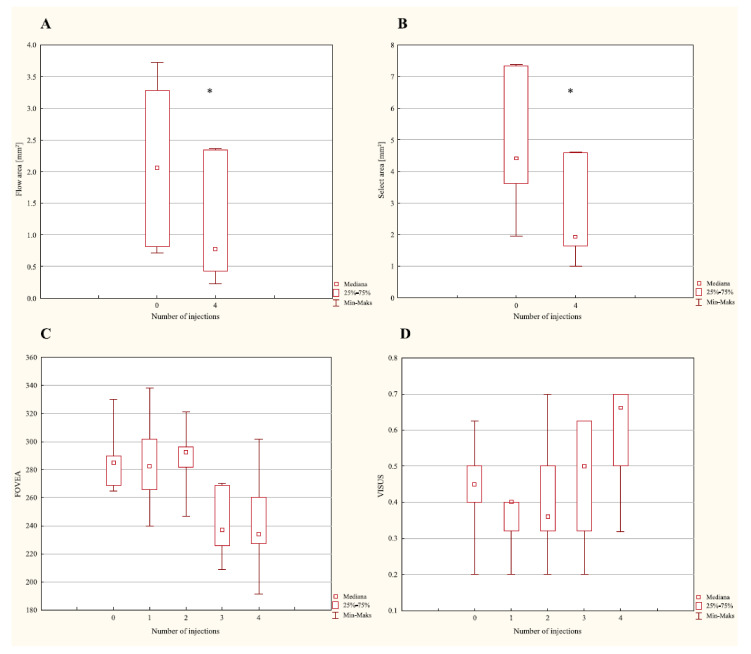
Changes in the values of parameters: flow area (**A**); select area (**B**); FOVEA (**C**); visus (**D**) among patients with AMD treated using brolucizumab (* *p* < 0.05).

The conducted statistical analysis indicated the incidence of statistically significant differences in the flow area, select area, FOVEA and visus parameters only in the group of patients, in whom brolucizumab was used (Table 1; Figure 2).

In patients treated with brolucizumab, a significant improvement in anatomical parameters was obtained, observed in the form of a significant reduction in intraretinal and subretinal fluid in the OCT examination and as a decrease in the size and activity of the CNV membrane in the OCTA examination.

In the OCT examination conducted before the first dose of brolucizumab was administered, the presence of intraretinal fluid in the sensory layer of the retina was determined, alongside subretinal and hyper-reflective deposits in the photoreceptor later, and segmental atrophy of the retinal pigment epithelium structure (RPE; Supplementary Materials Figure S1). After the first dose, a reduction in intraretinal and subretinal fluid was observed in the OCT (Supplementary Materials Figure S2), while, in macular OCT, after the injection of the third dose of brolucizumab, the disappearance of intraretinal fluid in the sensory layer was noted, as was a reduction in subretinal fluid (Supplementary Materials Figure S3). Whereas in macular OCT, after the injection of the fourth brolucizumab dose, the presence of intraretinal fluid in the sensory layer, alongside subretinal fluid, was not determined. Disturbances in the structure of the RPE layer were determined, additionally, there were also disappearances in the photoreceptor layer (Supplementary Materials Figure S4).

Before the first brolucizumab injection, in the OCTA examination, an active CNV membrane was determined in the outer layer of the retina and choriocapillaris. The presence of winding, dilated vessels with vascular loops was found around the perimeter of the membrane, with the lesion surrounded by a dark “halo” (Supplementary Materials Figure S5). On the other hand, after the first and third doses, a reduction in CNV membrane activity was determined, which was visible as a drop in the number of vascular loops and their tortuosity, as well as a reduction in the “halo” area surrounding the lesion (Supplementary Materials Figures S6 and S7). The OCT angiogram after the fourth brolucizumab injection indicated a lack of CNV activity, visible as dilated vessels with no “halo” area on the lesion periphery. An absence of a net of narrow vessels in the outer layer of the retina and choriocapillaris was observed (Supplementary Materials Figure S8).

Clinically, an improvement in visual acuity in patients treated using brolucizumab was noted. The obtained improvement in anatomical and functional parameters allowed for the extension of the interval between the next dose to 12 weeks (ANOVA Friedman; *p* = 0.0064).

**Figure 3:**
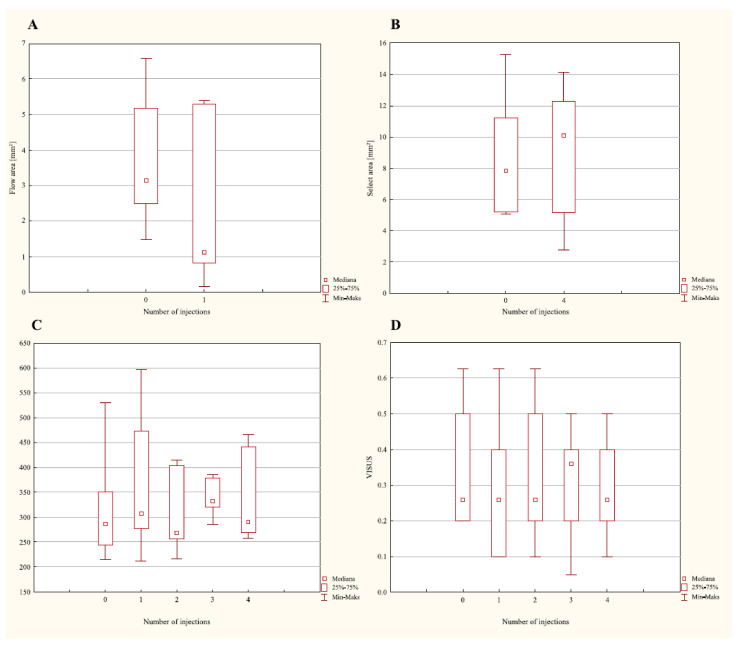
Changes in the values of the evaluated parameters: flow area (**A**); select area (**B**); FO-VEA (**C**); visus (**D**) in the group of patients with AMD treated using aflibercept.

However, no significant changes in the evaluated parameters among patients receiving aflibercept were determined (Table 2; Figure 3; *p* < 0.05). In patients treated using aflibercept, an insignificant reduction in intraretinal and subretinal fluid was achieved in the OCT examination, while in the OCTA examination, a reduction in the magnitude of change was noted after the initial three doses of the drug were administered 4 weeks apart.

In the OCT examination conducted before the first dose of aflibercept was administered, the presence of subretinal fluid and hyperreflective deposits between the outer and inner segments of the photoreceptors was determined. Disturbances and atrophy of the retinal pigment epithelium structure were also noted (RPE; Supplementary Materials Figure S9). After the first dose of the drug, an increase in the amount of subretinal fluid was noted (Supplementary Materials Figure S10), while after the injection of third and fourth dose, a reduction in subretinal fluid was not determined (Supplementary Materials Figures S11 and S12). In turn, after the injection of the fourth aflibercept dose, a reduction in subretinal fluid was noted, as well as disturbance in the retinal pigment epithelium structure with segmental atrophy and disturbance in the structure of the photoreceptor layer (Supplementary Materials Figure S13). An active CNV membrane was determined in the outer layer of the retina and choriocapillaris before the administration of the first dose, as well as after the first and third doses. The presence of winding, dilated vessels with vascular loops was found around the perimeter of the membrane, with the lesion surrounded by a dark “halo” (Supplementary Materials Figures S14–S16). A reduction in CNV membrane activity in the OCTA examination could only be determined after the fourth dose, visible as a drop in the number of vascular loops and their tortuosity in addition to a reduction in the “halo” area surrounding the lesion (Supplementary Materials Figures S17 and S18). The visual acuity in these patients remained unchanged or improved slightly. Following the administration of the fourth dose after 8 weeks, a small improvement in the anatomical and functional parameters was noted (ANOVA Friedman; *p* = 0.8781).

## 4. Discussion

Recently, in medicine, there has been a growing interest in understanding the molecular basis of diseases, which is a part of modern medicine. This has become possible thanks to the development of molecular biological techniques and methods [26]. The gold standard for treating the neovascular form of AMD is the use of VEGF inhibitors, which limit the activity of the diseases through the reduction of fluid in the retina and modification of its anatomical parameters, resulting in a long-term improvement or stabilization of visual acuity [27].

In our pilot study, we decided to compare the therapeutic response to anti-VEGF treatment in a group of patients with neovascular AMD treated with either brolucizumab or aflibercept. During the conducted observation, we noted only the expected therapeutic effect during brolucizumab treatment, with changes in flow area, select area and, FOVEA parameters being observable after the administration of the first dose of the drug already, and this effect maintained itself throughout the entire study. The “select area” is the measurement of the flow in the neovascular membrane itself, the boundaries of which are determined using the algorithm of the angio-OCT software (Optovue AngioVue^®^, Optovue, Inc.; Freemont, CA, USA). Higher values of this parameter in the aflibercept-treated group suggest a larger neovascular membrane during nAMD, however, the size of the neovascular membrane itself does not affect the treatment effectiveness of intravitreal injections. The possible structural damage to the retina is important, which directly affects the visual acuity achieved. Statistical analysis also confirmed favorable changes related to brolucizumab treatment, determined during OCT and OCT angiography examination (*p* < 0.05). Furthermore, an objective improvement in vision, calculated using visual acuity, was noted (*p* < 0.05). On the other hand, a significant improvement in the group of patients receiving aflibercept was not determined.

Research evaluating the effectiveness of both drugs in the indication of AMD has been conducted for a long time [28,29,30,31]. In Phase III of the double-blind HAWK clinical trial (NCT02307682), brolucizumab was used in patients with vascular AMD at a dose of 3 mg/50 µL (*n* = 358) or 6 mg/50 µL (*n* = 360), whereas aflibercept was used at a dose of 2 mg/50 µL (*n* = 360). An improvement in visual acuity in the group of patients receiving brolucizumab was noted, regardless of the dose, compared to the aflibercept-treated group (*p* < 0.001) [32,33,34]. In turn, in the HARRIER trial (NCT02434328), the patients received 6 mg/50 µL brolucizumab (*n* = 370) or 2 mg/50 µL aflibercept (*n* = 369), with an improvement in visual acuity being noted in the brolucizumab-treated group, compared to the aflibercept-treated group (*p* < 0.001) [25,35,36]. Bullrish et al. [37] assessed brolucizumab treatment in 57 patients with neovascular AMD, who had previously undergone a different form of anti-VEGF treatment (ranibizumab, aflibercept, and bevacizumab). They noted a change in BVA (−0.03 ± 0.14), logMAR (*p* = 0.115), a reduction in FCP (−66.81 ± 72.643 µm; *p* < 0.001), CSRT (−66.76 ± 60.71 µm; *p* < 0.001) and spot volume (0.27 ± 0.24 mm^3^; *p* < 0.001). Ultimately, these authors determined that bevacizumab is a promising form of AMD therapy in patients, in whom an adequate response to treatment was not achieved using other VEGF inhibitors [37]. Observing drug resistance during molecular target therapies (anti-cytokine therapies) is nothing new, with this situation being observed in oncology, dermatology, and ophthalmology [38,39]. The observed difference in effectiveness between therapies based on aflibercept and brolucizumab is most likely the result of the different structures of the two inhibitors, the size of the molecule as well as the differing influence on signaling pathways dependent on VEGF. Garweg, in his study, indicates that one of the potential reasons for the higher effectiveness of brolucizumab, compared with aflibercept, stems from the fact that the former VEGF inhibitor achieves an approximately 12-times higher equivalent molar dose [40]. The advantage of brolucizumab, in comparison to aflibercept, was also indicated in the double-blind multicenter second phase trial conducted on a group of 89 patients with active choroidal neovascularization secondary to AMD, who were previously untreated. Higher stability in the changes during AMD in the patients treated using brolucizumab was observed, resulting in a smaller number of unscheduled surgeries (6 vs. 15). Additionally, it was observed that, compared to aflibercept, a larger number of patients treated with brolucizumab noted that issues with intraretinal and subretinal fluid were resolved. At the same time, the safety of pharmacotherapy was confirmed, with the use of both anti-VEGF drugs [41].

Furthermore, it is worth considering that VEGF is continuously synthesized. In turn, the therapeutic effect, and its duration also, are dependent on the ability to inhibit VEGF through the drug administered to the vitreous [42,43].

Observations varying from ours were made by Eissing et al. [44], who using mathematical analysis, which in turn, was based on a pharmacodynamic and pharmacokinetic profile, assessed the ability of the three drugs, namely aflibercept, ranibizumab, and brolucizumab, to reduce VEGF concentration. In summation, these authors determined that, considering the average half-life duration of brolucizumab, its use in the case of AMD can be insufficient to inhibit VEGF expression when used every 12 weeks, in comparison to aflibercept [44].

Directly after drug administration, VEGF expression is fully inhibited as the drug is administered at a molar concentration greater than that of VEGF [45]. Thus, as the molar mass of the drug increases, the length of its half-life becomes greater (aflibercept 97–155 kDa > ranibizumab 48 kDa > brolucizumab 26 kDa) [44]. Moreover, the effectiveness is dependent on the affinity of the inhibitor to VEGF (aflibercept > brolucizumab > ranibizumab) [46]. Discrepancies can also result from the fact that brolucizumab is a relatively recently introduced VEGF inhibitor, with research on the use and safety of therapy still ongoing [42,43]. Maloney et al. [45], however, based on meta-analysis, did not determine that the use of any of the three VEGF inhibitors: aflibercept; brolucizumab; ranibizumab was related to a heightened risk of acute cerebrovascular disease, major bleeding, myocardial infarction, or all-cause hospitalization [45,46].

Furthermore, as highlighted by the research conducted by Mitchel et al. [47], they indicate that people with nAMD, in whom central vision is disturbed, struggle with anxiety, depression, and the need to rely on another person, which in turn, negatively impacts their mental state [47]. The stress level amongst patients with nAMD with a visual acuity of 20/200 is comparable to that felt by patients with human immunodeficiency virus and melanoma [48]. In the context of previous findings [41,42,43,44], as well as the preliminary observations described in this study, it is worth paying attention to the study conducted by Fernandes et al. [49], who indicate that the low molar mass of brolucizumab, compared to the two aforementioned inhibitors, is the factor which conditions such positive therapeutic effects. This contributes to better penetration of the drug molecule into the tissue, higher local effectiveness, a longer durability of the therapeutic effect, and a lower risk of a systemic side effect occurring, compared with a full-sized immune-globulin G [50]. Additionally, thanks to the excellent solubility of brolucizumab, it can be concentrated to 120 mg/mL, allowing for the one-time administration of 6 mg/50 µL in a single injection. At this concentration and injection volume, enough brolucizumab molecules are delivered, insomuch that the binding capacity for VEGF inactivation is 11 and 22 times greater than for aflibercept and ranibizumab, respectively. It seems possible that this is therapeutically advantageous, due to the prolonged action of the drug [50].

Of course, our research also has weaknesses, which limit it. Firstly, this is a single-center, pilot study carried out on a group of 12 patients with nAMD. Moreover, the observation period lasted around 20/24 weeks, depending on the drug, and does not allow us to formulate long-term conclusions regarding the effects of brolucizumab and aflibercept therapy. Due to the dynamic development of new treatment methods in ophthalmology, our observations are significant [51,52], but they do require further research.

## 5. Conclusions

In summary, brolucizumab therapy may be more effective, compared to aflibercept therapy, in patients with nAMD during an observation period of around 20/24 weeks. Despite the limitations of the research, our observations are significant [51,52], but they do require further research.

## Figures and Tables

**Table 1:** Changes in the values of the evaluated parameters in the group of patients with AMD treated using brolucizumab.

Parameters	Number of Injections	Me	Q1	Q3	*p* < 0.05
Flow area [mm^2^]	0	2.0615	0.8140	3.2760	0.0277 (Wilcoxon test)
	4	0.7855	0.4250	2.3450	
Select area [mm^2^]	0	4.4135	3.6150	7.3540	0.0277 (Wilcoxon test)
	4	1.9345	1.6570	4.6010	
FOVEA	0	285.5000	269.0000	290.0000	0.0073 (ANOVA Friedman analysis)
	1	282.5000	266.0000	302.0000	
	2	292.5000	282.0000	296.0000	
	3	237.0000	226.0000	269.0000	
	4	234.0000	227.0000	260.0000	
Visus	0	0.4500	0.4000	0.5000	0.0064 (ANOVA Friedman analysis)
	1	0.4000	0.3000	0.4000	
	2	0.3500	0.3000	0.5000	
	3	0.5000	0.3000	0.5000	
	4	0.5000	0.3000	0.6250	

Me—Median; Q1—lower quartile; Q3—upper quartile.

**Table 2:** Changes in the values of the evaluated parameters in the group of patients with AMD treated using aflibercept.

Parameters	Number of Injections	Me	Q1	Q3	*p* < 0.05
Flow area [mm^2^]	0	3.1395	2.4800	5.1780	0.3454 (Wilcoxon test)
	4	1.1270	0.8410	5.2890	
Select area [mm^2^]	0	7.8530	5.2010	11.2440	0.7532 (Wilcoxon test)
	4	10.1060	5.1440	12.2500	
FOVEA	0	287.0000	245.0000	350.0000	0.6626 (ANOVA Friedman analysis)
	1	308.0000	278.0000	473.0000	
	2	269.0000	257.0000	403.0000	
	3	332.0000	321.0000	378.0000	
	4	291.5000	269.0000	441.0000	
Visus	0	0.2600	0.2000	0.5000	0.8781 (ANOVA Friedman analysis)
	1	0.2600	0.1000	0.4000	
	2	0.2600	0.2000	0.5000	
	3	0.3600	0.2000	0.4000	
	4	0.2600	0.2000	0.4000	

Me—Median; Q1—down quartile; Q3—upper quartile.

## Data Availability

The data used to support the findings of this study are included in the article.

## Supplementary Materials

The following are available online at https://www.mdpi.com/article/10.3390/ijerph19042303/s1, Figure S1. Macular OCT prior to injection of brolucizumab. Figure S2. Macular OCT after injection of the first dose of brolucizumab. Figure S3. Macular OCT after injection of the third dose of brolucizumab. Figure S4. Macular OCT after fourth injection of brolucizumab. Figure S5. OCT angiogram before injection of brolucizumab. Figure S6. OCT angiogram after injection of the first dose of brolucizumab. Figure S7. OCT angiogram after injection of the third dose of brolucizumab. Figure S8. OCT angiogram after fourth injection of brolucizumab. Figure S9. OCT before the injection of aflibercept. Figure S10. Macular OCT after injection of the first dose of aflibercept. Figure S11. Macular OCT after the second injection of the aflibercept dose. Figure S12. Macular OCT after the third injection of the aflibercept dose. Figure S13. Macular OCT after fourth dose of aflibercept injection. Figure S14. OCT angiogram before first injection of brolucizumab. Figure S15. OCT angiogram after first injection of aflibercept. Figure S16. OCT angiogram after the third injection of aflibercept. Figure S17. OCT angiogram after fourth injection of aflibercept. Figure S18. OCTA after the 6th injection of aflibercept.
