# Supplementary material for: The Effectiveness of Brolucizumab and Aflibercept in Patients with Neovascular Age-Related Macular Degeneration
Source: Int J Environ Res Public Health. 2022 Feb 17;19(4):2303. doi: 10.3390/ijerph19042303 (PMC8872595; doi:10.3390/ijerph19042303)
Supplement: Supplementary file 1 [file ijerph-19-02303-s001.zip › ijerph-1548496-supplementary.pdf]

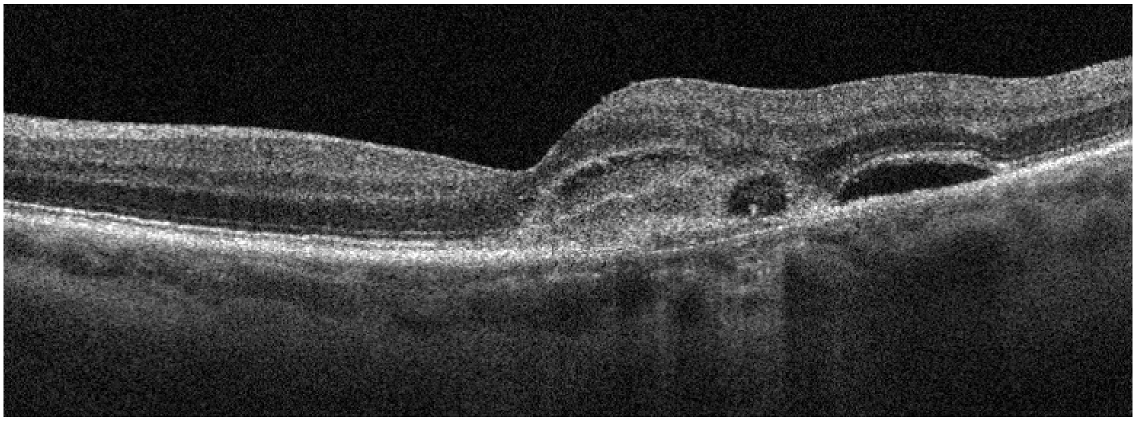

Figure S1. Macular OCT prior to injection of brolucizumab.

In the OCT examination conducted before the first dose of brolucizumab was administered, the presence of intraretinal fluid in the sensory layer of the retina was determined, as well as subretinal and hyperreflective deposits in the photoreceptor layer, and segmental atrophy of the structure of the retinal pigment epithelium (RPE).

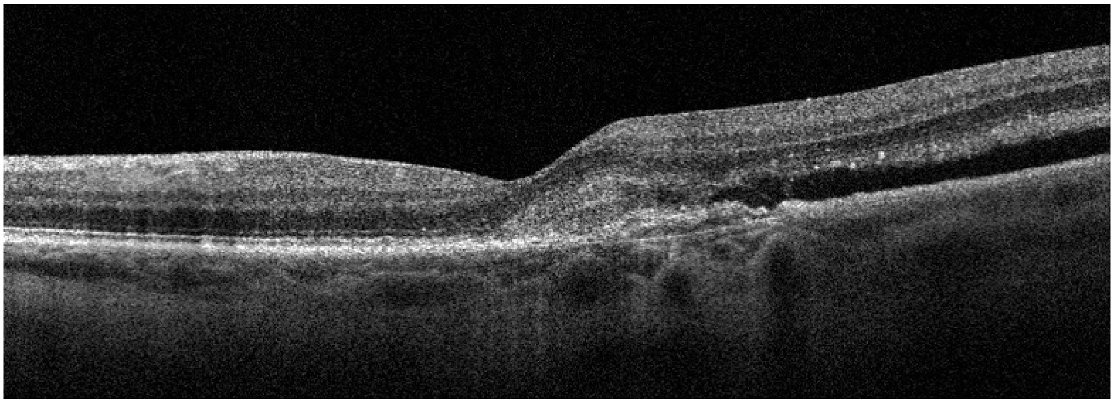

Figure S2. Macular OCT after injection of the first dose of brolucizumab

After the first dose, a reduction in intraretinal and subretinal fluid was observed in the OCT examination.

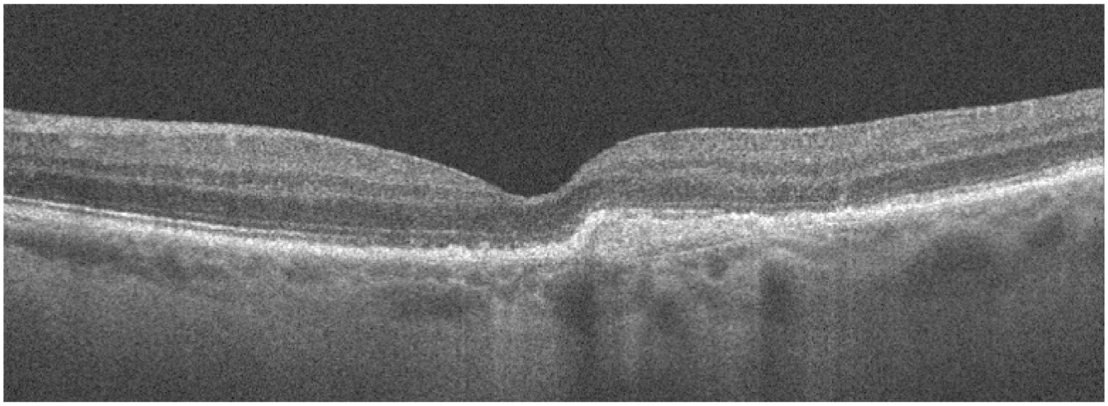

Figure S3. Macular OCT after injection of the third dose of brolucizumab

After the injection of a third dose of brolucizumab, the disappearance of intraretinal fluid in the sensory layer was noted with a reduction in subretinal fluid also.

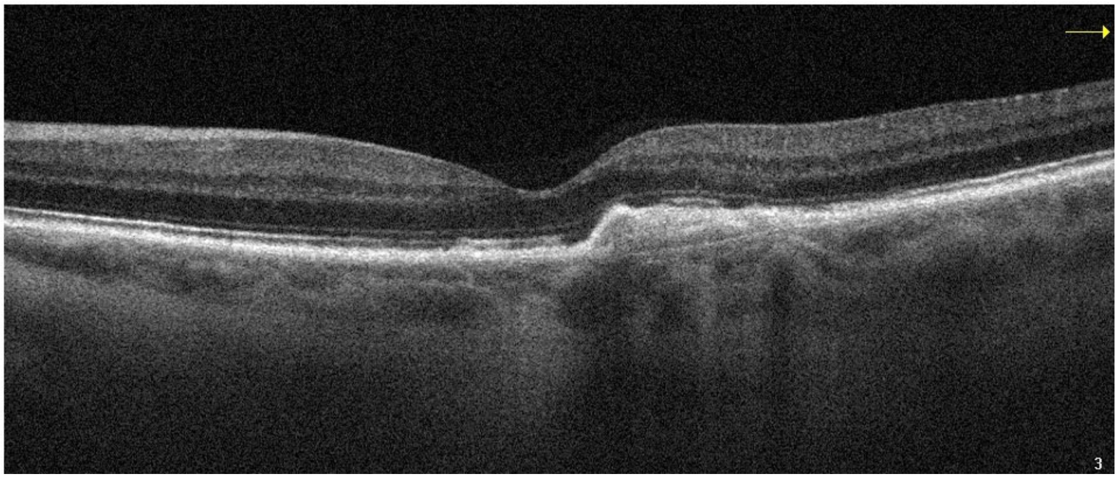

Figure S4. Macular OCT after the fourth injection of brolucizumab
Disturbances in the structure of the RPE layer were determined, and also disappearances in the photoreceptor later.

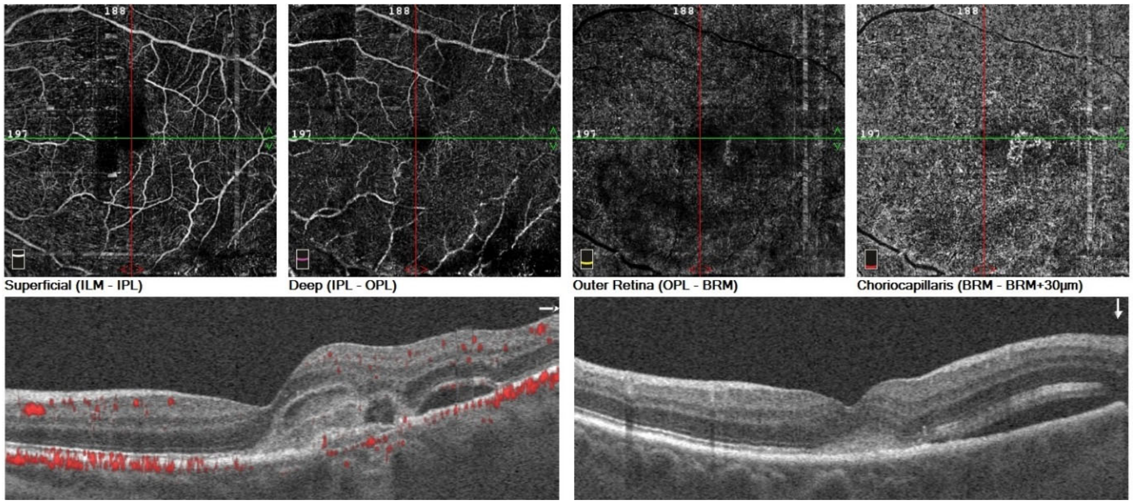

Figure S5. OCT angiogram before injection of brolucizumab

Winding, dilated vessels with vascular loops around the perimeter of the membrane were found, the lesion is surrounded by a dark “halo”.

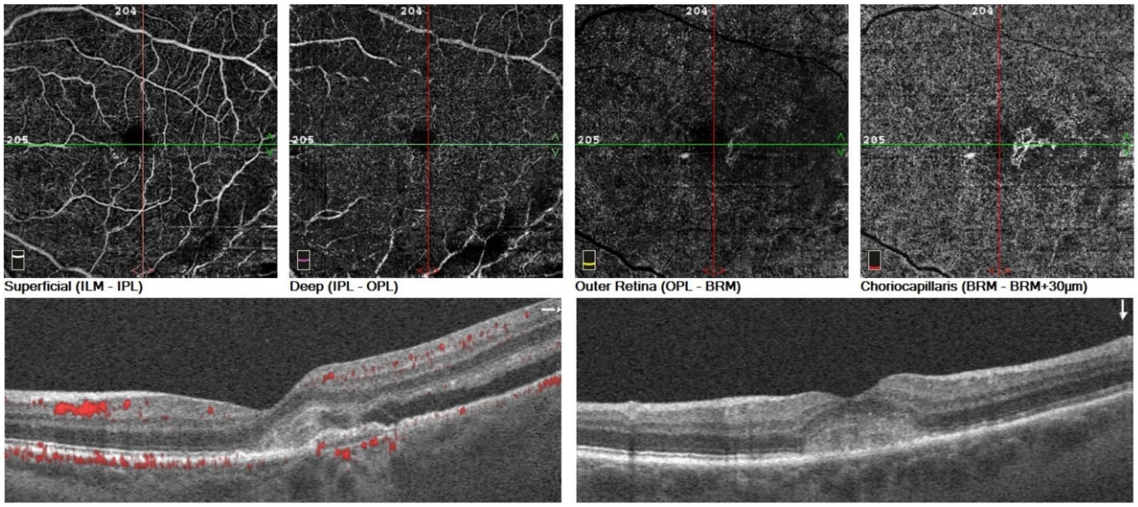

Figure S6. OCT angiogram after injection of the first dose of brolucizumab

After the first and third dose of the drug, a reduction in CNV membrane activity was determined, which was visible as a drop in the number of vascular loops and their tortuosity as well as a reduction in the "halo" area surrounding the lesion.

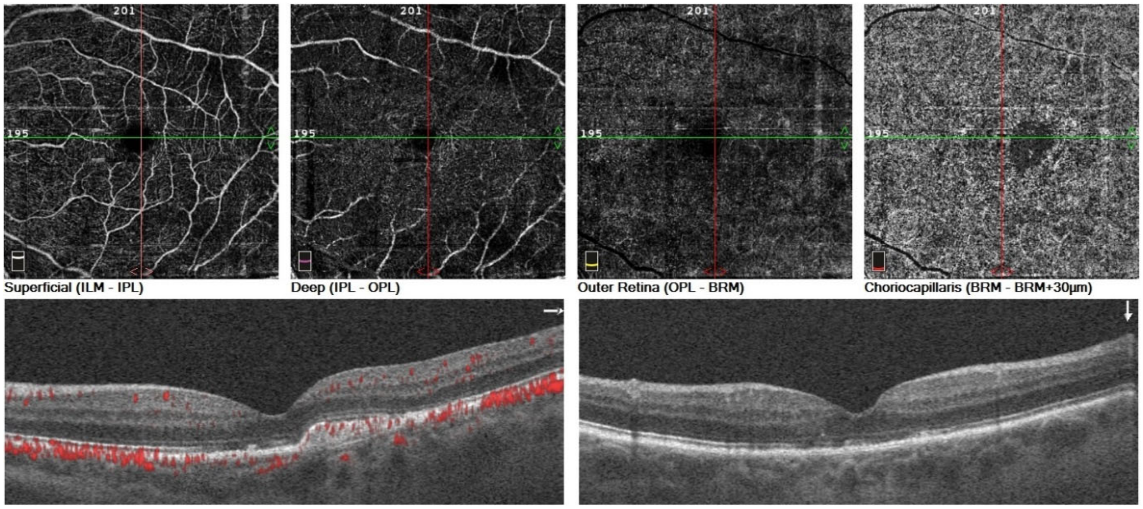

Figure S7. OCT angiogram after injection of the third dose of brolucizumab
After the first and third dose of the drug, a reduction in CNV membrane activity was determined, which was visible as a drop in the number of vascular loops and their tortuosity and a reduction in the "halo" area surrounding the lesion.

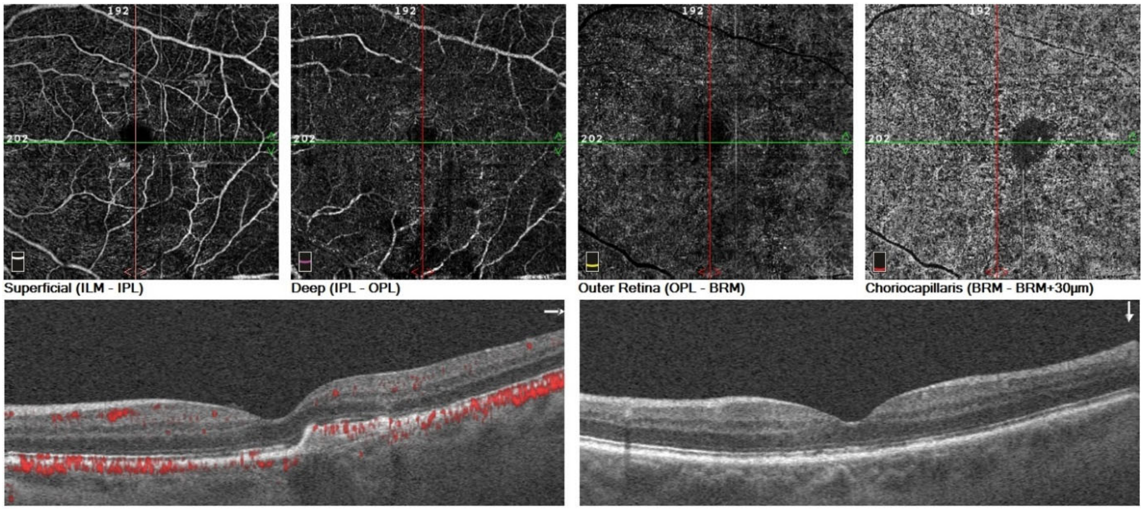

Figure S8. OCT angiogram after the fourth injection of brolucizumab
After the fourth injection of brolucizumab, a lack of CNV activity was indicated, visible as dilated vessels with no “halo” area on the periphery of the lesion. Additionally, there was an absence of a net of narrow vessels in the outer layer of the retina and choriocapillaris.

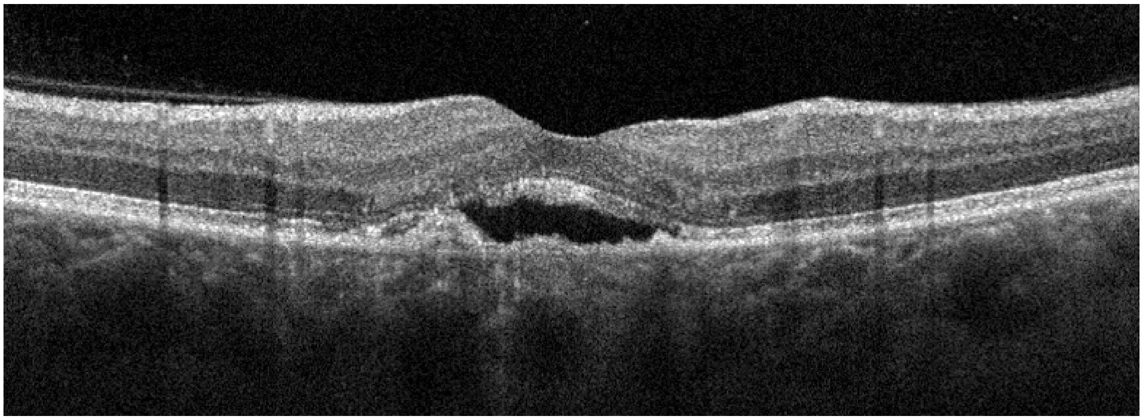

Figure S9. OCT before the injection of aflibercept
The presence of subretinal fluid and hyperreflective deposits was determined between the outer and inner segments of the photoreceptors. Disturbances and atrophy of the structure of the retinal pigment epithelium were also noted.

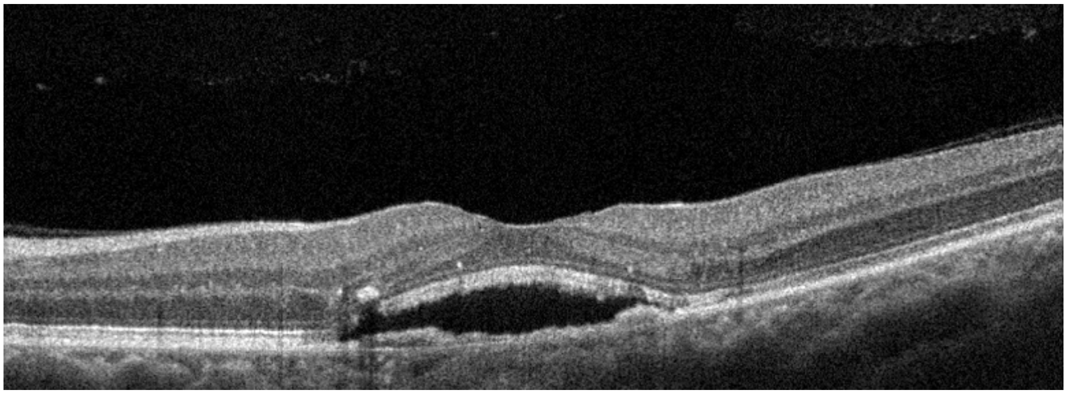

Figure S10. Macular OCT after injection of the first dose of aflibercept
After the first dose of the drug, an increase in the amount of subretinal fluid was noted.

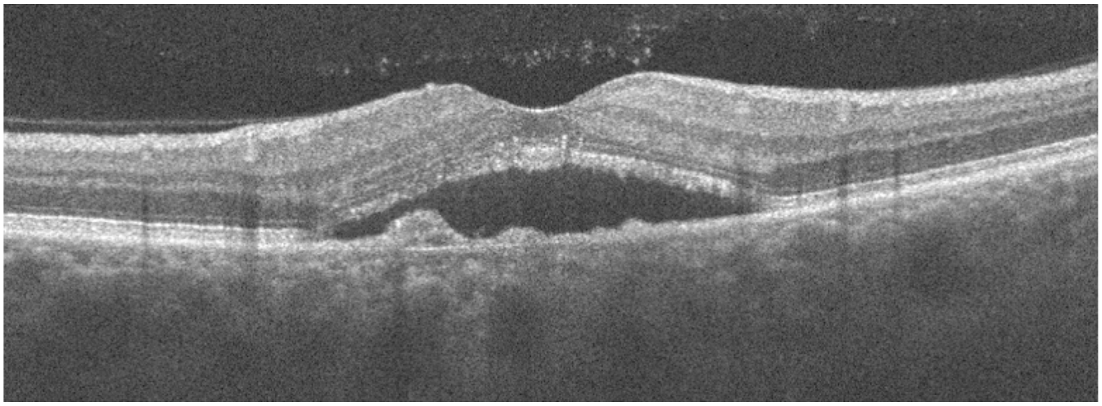

Figure S11. Macular OCT after the second injection of the aflibercept dose
After the injection of the third and fourth dose, a reduction in subretinal fluid was not determined.

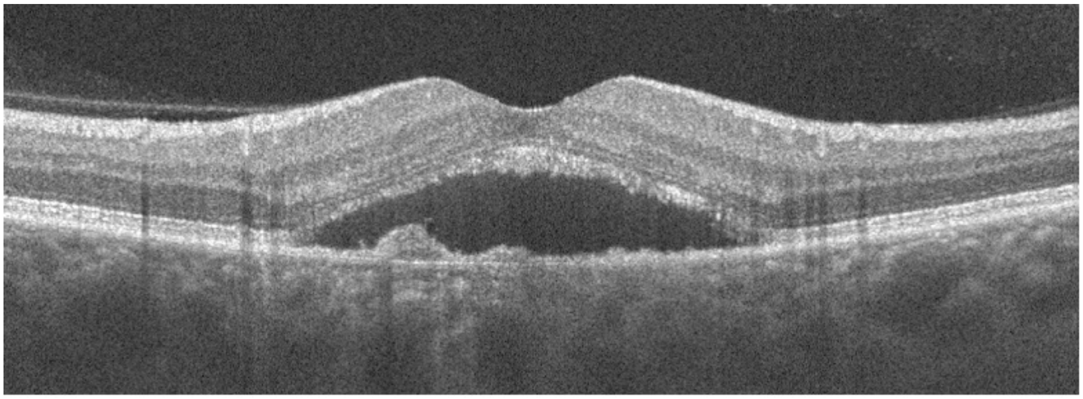

Figure S12. Macular OCT after the third injection of the aflibercept dose
After the injection of the third and fourth dose, a reduction in subretinal fluid was not determined.

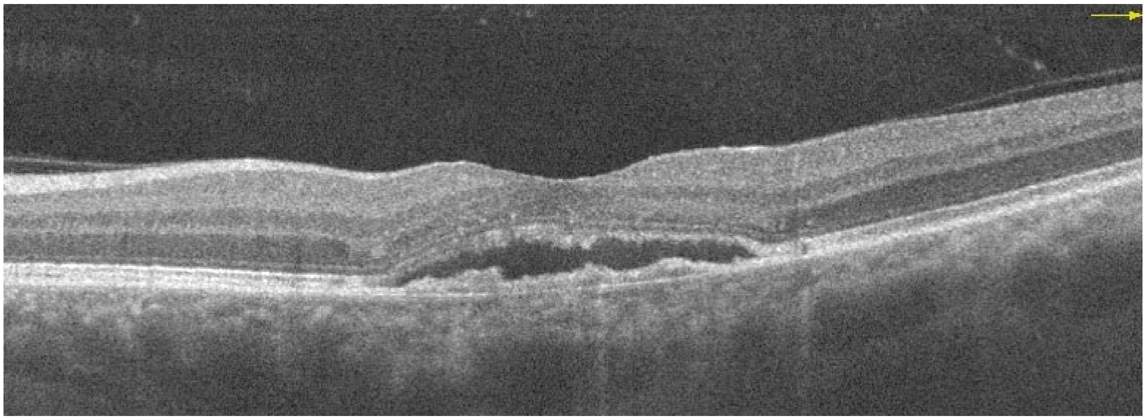

Figure S13. Macular OCT after fourth dose of aflibercept injection
After the injection of the fourth dose of aflibercept, a reduction in the subretinal fluid was noted, in addition to disturbance in the structure of the retinal pigment epithelium with segmental atrophy as well as disturbance in the structure of the photoreceptor layer.

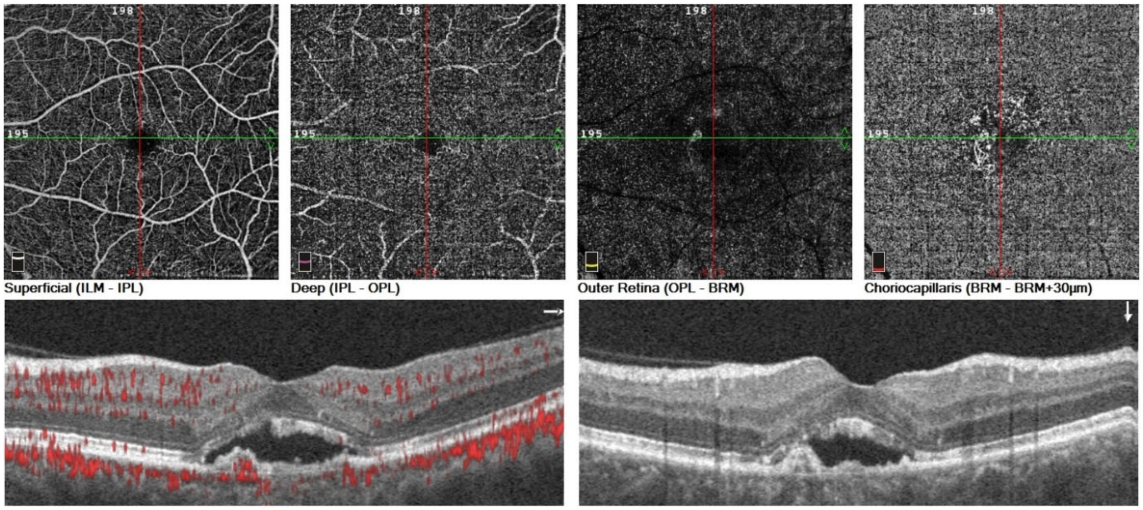

Figure S14. OCT angiogram before first injection of brolucizumab.
Before the administration of the first dose of the drug, an active CNV membrane was determined in the outer layer of the retina and choriocapillaris, containing winding, dilated vessels with vascular loops around the perimeter of the membrane, the lesion is surrounded by a dark “halo”

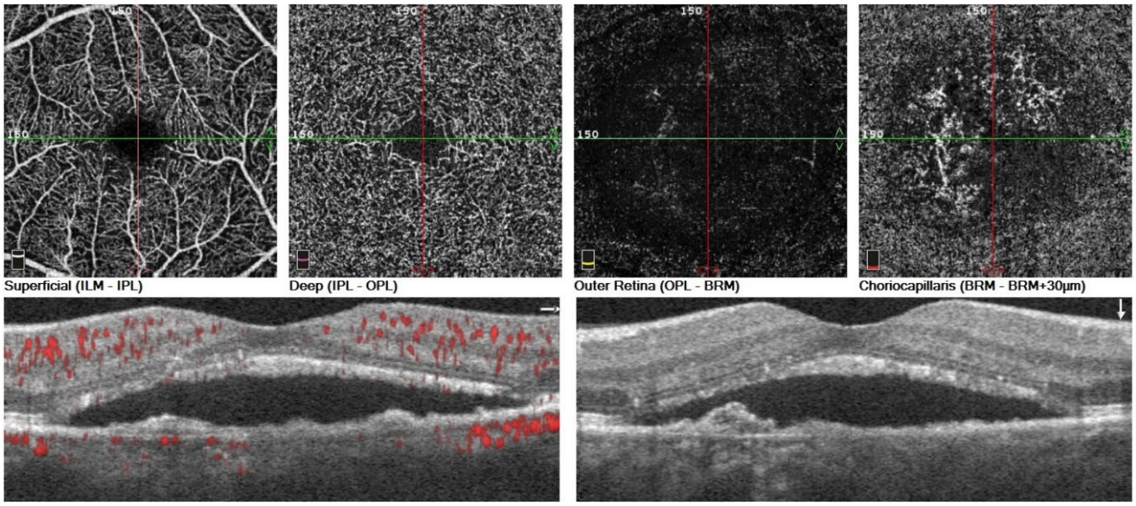

Figure S15. OCT angiogram after the first injection of aflibercept.
After the first dose, an active CNV membrane was determined in the outer layer of the retina and choriocapillaris. Winding, dilated vessels with vascular loops were found around the perimeter of the membrane, the lesion is surrounded by a dark “halo”

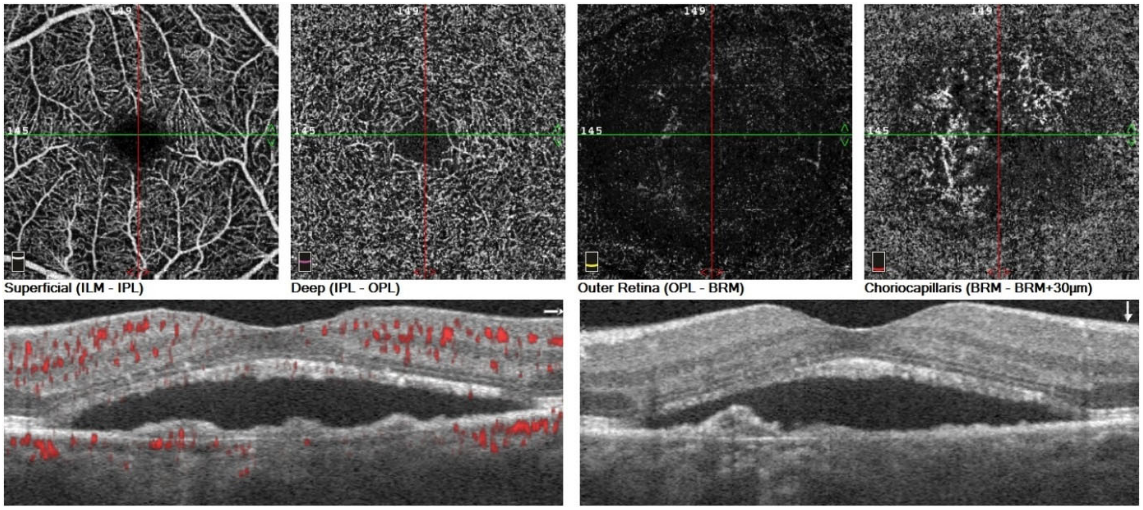

Figure S16. OCT angiogram after the third injection of aflibercept

After the third dose, an active CNV membrane was determined in the outer layer of the retina and choriocapillaris. The presence of winding, dilated vessels with vascular loops was determined around the perimeter of the membrane, the lesion is surrounded by a dark “halo”.

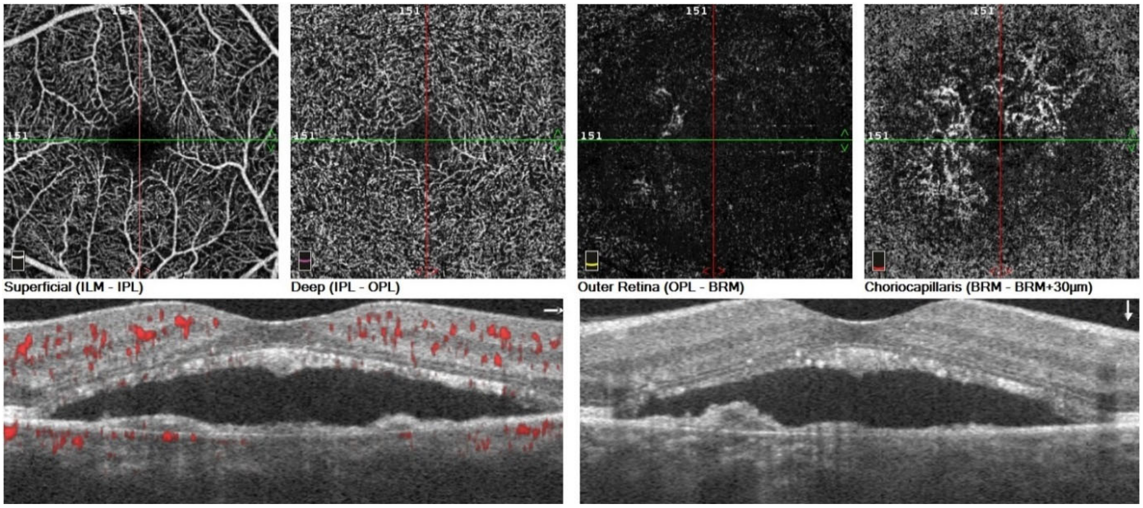

Figure S17. OCT angiogram after fourth injection of aflibercept

After the fourth dose, it was possible to determine a reduction in CNV membrane activity in the OCTA examination, which was visible as a drop in the number of vascular loops and their tortuosity in addition there was a reduction in the “halo” area surrounding the lesion also.

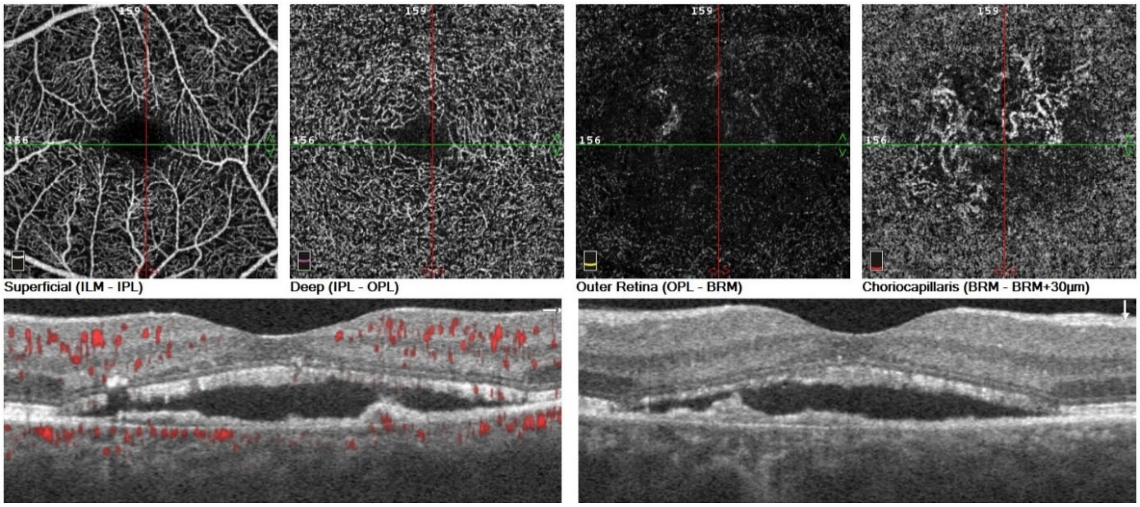

Figure S18. OCT angiogram after the 6th injection of aflibercept

After the fourth dose, it was possible to determine a reduction in CNV membrane activity in the OCTA examination, which was visible as a drop in the number of vascular loops and their tortuosity as well as a reduction in the “halo” area surrounding the lesion
